# Unmasking the Silent Threat: Periodontal Health’s Impact on COPD Severity and Hospitalization

**DOI:** 10.3390/jpm13121714

**Published:** 2023-12-15

**Authors:** Anitha Subbappa, Komarla Sundararaja Lokesh, Sindaghatta Krishnarao Chaya, Mohammed Kaleem Ullah, Jayaraj Biligere Siddaiah, Nandlal Bhojraj, Padukudru Anand Mahesh

**Affiliations:** 1 Department of Periodontology, JSS Dental College & Hospital, JSS Academy of Higher Education and Research, Mysuru 570015, India; dr.anithas@jssuni.edu.in; 2 Department of Respiratory Medicine, JSS Medical College, JSS Academy of Higher Education and Research, Mysuru 570015, India; kslokesh@jssuni.edu.in (K.S.L.); skchaya@jssuni.edu.in (S.K.C.); bsjayaraj@jssuni.edu.in (J.B.S.); 3 Centre for Excellence in Molecular Biology and Regenerative Medicine (A DST-FIST Supported Center), Department of Biochemistry (A DST-FIST Supported Department), JSS Medical College, JSS Academy of Higher Education and Research, Mysore 570015, India; ka7eem@jssuni.edu.in; 4 Division of Infectious Disease and Vaccinology, School of Public Health, University of California, Berkeley, CA 94720, USA; 5 Special Interest Group—Environment and Respiratory Diseases, JSS Academy of Higher Education and Research, Mysuru 570015, India

**Keywords:** COPD, periodontitis, pulmonary function tests

## Abstract

Objective: This study investigated the relationship between chronic obstructive pulmonary disease (COPD) and periodontitis, focusing on how periodontal health impacts COPD airflow limitation, exacerbations, and hospitalization. Background: Periodontitis, a multifactorial inflammatory disease, is characterized by destruction of tooth-supporting structures, while COPD is a global pulmonary disorder with high mortality. Methods: A total of 199 COPD patients aged over 40 years underwent lung function tests (spirometry), 6 min walk test, and St George’s Respiratory Questionnaire-COPD (SGRQ-C) to assess lung health. Periodontal indices such as probing depth (PD), clinical attachment loss (CAL), and plaque index (PI) were assessed. Results: We found a significant negative correlation between periodontal disease severity and lung function (lower FEV1, FVC, and FEV1/FVC ratio) after adjusting for smoking. Likewise, periodontal parameters (PPD, PI, and CAL) exhibited negative correlations with lung function. These periodontal indices were independently associated with airflow limitation severity, exacerbations frequency, and prior-year hospitalization. Linear regression indicated that each unit increase in PPD, PI, and CAL corresponded to estimated increases in GOLD airflow limitation grading (0.288, 0.718, and 0.193, respectively) and number of exacerbations (0.115, 0.041, and 0.109, respectively). In logistic regression, PPD, PI, and CAL adjusted odds ratios (ORs) were estimated to increase by 1.29 (95%CI: 1.03–1.62), 3.04 (95%CI: 1.28–7.2), and 1.26 (95%CI: 1.06–1.49), respectively, for hospitalization in previous year. Conclusion: Periodontitis is associated with COPD airflow limitation, exacerbation, and hospitalization, with PI being the most clinically relevant periodontal factor. Dentists and physicians should monitor and increase awareness among COPD patients to maintain oral hygiene for prevention of periodontal diseases and mitigate its effect on COPD progression.

## 1. Introduction

Periodontitis, a chronic inflammatory disease impacting the periodontium and often resulting in tooth loss, primarily stems from host–microbial interactions. While these interactions are pivotal in initiating and advancing the disease, numerous multifactorial etiological factors, encompassing local and systemic elements, environmental influences, and genetic factors, contribute to the exacerbation of periodontitis [1,2]. Recent scientific evidence sheds light on the link between untreated periodontitis and systemic health issues, including diabetes, adverse birth outcomes, and cardiovascular disease [3]. This connection is further elucidated by understanding that systemic, environmental, and psychological factors, while not directly causing periodontitis, modify the host’s inflammatory response, leading to changes in vascular and cellular responses that predispose toward or accelerate the destruction of periodontal tissue [4]. 

Importantly, there are no distinct periodontal features exclusive to diabetic patients with periodontitis compared to those with periodontitis alone. However, persistent uncontrolled hyperglycemia emerges as a significant factor influencing periodontitis progression [5,6,7]. Moreover, diabetes is established as a risk factor for gingivitis and periodontitis, with the level of glycemic control serving as a crucial determinant in this association [8]. Pregnant women with active periodontal diseases are identified as being at risk for adverse pregnancy outcomes (APOs), encompassing preterm birth (PTB), fetal growth restriction, low birth weight, pre-eclampsia, and gestational diabetes [9]. The biological interaction linking periodontitis to systemic inflammation, through the translocation of oral microbiota impacting atherothrombogenesis, underscores the intricate connection between periodontal diseases and cardiovascular disease [10]. Moreover, there is a growing understanding of how systemic disorders can impact periodontitis in return [11]. 

The intricate interplay between oral infections and secondary systemic effects is mediated through several potential mechanisms or pathways. These include the metastatic spread of oral infection via transient bacteremia, the adverse effects of circulating oral microbial toxins, and the induction of metastatic inflammation through immunological down-regulation by oral microorganisms [12,13,14]. This emerging knowledge underscores the significance of exploring the relationship between periodontitis and various systemic diseases to better comprehend their interconnectedness and potential shared pathophysiology.

Chronic obstructive pulmonary disease (COPD) is one of the most prevalent respiratory conditions, characterized by progressive, irreversible airflow obstruction, emphysema, and fibrosis, culminating in diminished lung function and increased mortality rates [15,16,17]. The alarmingly high prevalence of COPD, the presence of symptoms such as cough, sputum production, and dyspnea due to restricted airflow, accelerated progression of disease due to frequent exacerbations, and low quality of life has made COPD one of the important leading causes of morbidity and mortality, resulting in more than 3 million deaths worldwide [18,19]. Addressing modifiable risk factors is crucial to slowing down COPD progression, where tobacco smoking, biomass fuel smoke, air pollution, occupational exposure, and poor nutrition are commonly addressed risk factors. Given the anatomical continuity between the oral cavity and the respiratory system, the oral cavity holds the potential to act as a reservoir for respiratory pathogens, making good oral health crucial and often neglected [20].

Consequently, researchers have investigated the association between periodontitis and COPD, with promising outcomes, indicating that periodontal therapy can reduce COPD exacerbation [21]. A systematic review by Apessos et al. observed a slower reduction in lung function, decreased frequency of COPD exacerbation, lower hospitalization rates, and reduced all-cause mortality in COPD patients with periodontitis undergoing periodontal therapy [21]. These findings have sparked interest in understanding the underlying mechanisms connecting these seemingly distinct diseases. 

In individuals with periodontitis, bacteria in periodontal pockets might enter the bloodstream, with aspiration of oropharyngeal secretions serving as the most common route for these pathogens to reach the lungs [22]. While the impact of periodontitis on COPD has been studied in Western countries such as those in Europe and the United States of America, its specific implications in the Indian population remain largely unexplored. This study aimed to evaluate the relationship between periodontitis and COPD severity, exacerbations, and hospitalizations. Understanding this association could have profound implications for both dental and respiratory healthcare professionals, urging collaboration to enhance patient care and overall well-being.

## 2. Materials and Methods

### 2.1. Study Design and Patients

This cross-sectional study involved 199 spirometry-confirmed COPD subjects enrolled from the Mysuru Studies of Determinants of Health in Rural Adults (MUDHRA) Cohort [23] in the Mysuru district., involving subjects from 16 villages (8 villages from Mysuru Sub-district and 8 villages from Nanjangud Sub-district). The details of the MUDHRA cohort have been previously published [23]. The study was conducted by the Department of Respiratory Medicine and Department of Periodontology, JSS Hospital, a tertiary care university teaching hospital, in Mysuru, India. Written informed consent was obtained from all the subjects. The study was approved by the Institutional Ethics Committee of JSS Medical College, Mysuru (Approval number: JSSMC/IEC/15/2007/2017-18 Dated 12 October 2017). 

### 2.2. Inclusion and Exclusion Criteria

Spirometry-confirmed male COPD patients aged 40 or older, smokers, with more than 10 teeth who were willing to participate, were screened. Patients with a history of periodontal therapy in the past 6 months, pregnancy, inflammatory diseases other than COPD, cancer, HIV infection, or previous lung surgery, and subjects unable to perform spirometry or having a contraindication for spirometry and six-minute walk test, were excluded from the study.

### 2.3. Data Collection

An interview utilizing a questionnaire was conducted which included demographic variables such as age, sex, marital status, occupation, educational level, living condition, smoking status, smoking pack-years, previous exacerbation, hospitalization, and St George’s respiratory questionnaire-C (SGRQ-C). Lung function testing, specifically Forced Vital Capacity (FVC) using spirometry, was conducted according to American Thoracic Society (ATS) and Global Initiative for Chronic Obstructive Lung Disease (GOLD) guidelines utilizing an ndd Easyone proTM Lab device. Furthermore, exercise capacity was assessed via a six-minute walk test performed according to ATS guidelines.

### 2.4. Spirometry

The post-bronchodilator values are presented as percentages of forced vital capacity (FVC % Pred), forced expiratory volume in 1 **s** (FEV_1_ % Pred) over predicted value, and the ratio of FEV_1_/forced vital capacity (FEV_1_/FVC). The confirmation of COPD and grading the severity of airflow limitation were conducted according to GOLD guidelines: Grade I (mild: FEV_1_/FVC < 0.70, and FEV_1_ ≥ 80% predicted), Grade II (moderate: FEV_1_/FVC < 0.70 and FEV_1_ from 50% to 80% predicted), Grade III (severe: FEV_1_/FVC < 0.70 and FEV_1_ from 30% to 50% predicted), and Grade IV (very severe: FEV_1_/FVC < 0.70 and FEV_1_ < 30% predicted) [24].

### 2.5. Evaluation of Periodontal Status

During the oral examinations, six sites on each tooth were examined utilizing the UNC-15 periodontal probe. Probing pocket depth (PPD), plaque index (PI), and clinical attachment loss (CAL) were assessed for every tooth, and subsequently, an average value was calculated.

### 2.6. Assessment of Probing Pocket Depth and Clinical Attachment Levels

The measurement of probing pocket depth (PPD) was conducted by assessing the distance between the gingival edge and the base of the periodontal pocket. The measurement of clinical attachment level (CAL) involved assessing the distance between the cementoenamel junction and the base of the periodontal pocket. The PPD (probing pocket depth) and CAL (clinical attachment level) measurements were manually recorded using a University of North Carolina-15 (UNC-15) probe and a mouth mirror. The measurement of the PPD (probing pocket depth) and CAL (clinical attachment level) was conducted using a calibrated UNC-15 periodontal probe. The probe is 15 millimeters in length and is equipped with millimeter markings at each millimeter, as well as color labeling at the 5th-, 10th-, and 15th-millimeter intervals. The probe is inserted with a firm, gentle pressure to the bottom of the pocket. Each tooth underwent examination at four specific areas, specifically the mid-facial, mesio-facial, disto-facial, and center of lingual surfaces [25,26]. The diagnosis of periodontal disease was made based on the classification suggested at the 2018 World Workshop on the Classification of Periodontal and Peri-Implant Diseases and Conditions, as outlined below: when examining the inter-dental clinical attachment level (CAL) of two teeth that are not contiguous, or the buccal or oral CAL, it was observed that the CAL measurement was equal to or greater than 3 mm, accompanied by pocketing exceeding 3 mm (Tonnetti) [27].

### 2.7. Assessment of Plaque Index (PI)

We utilized the Silness–Löe plaque index to evaluate oral hygiene [28]. This assessment method entails the examination of soft debris and mineralized deposits on specific teeth while excluding missing teeth from the evaluation. Each tooth’s four surfaces (buccal, lingual, mesial, and distal) were individually scored within a range of 0 to 3.

### 2.8. Statistical Analysis

The data were analyzed using IBM SPSS Statistics 22 and Jamovi 2.3. Numerical variables are presented as either mean ± SD or median (IQR), while the categorical variables are displayed as frequencies and percentages. ANOVA, chi-square, and Kruskal–Wallis tests were used in relation to the variables. Additionally, the variables PPD, PI, and CAL were compared across the GOLD grades of airflow limitation and exacerbation numbers. The regression analyses were performed to evaluate the association of various factors. Linear regression plots for lung function parameters (post-bronchodilator FEV_1_ percent predicted, FVC percent predicted, and FEV_1_/FVC ratio), periodontitis severity, and periodontal parameters (PPD, PI, and CAL) with adjustment for smoking. Multiple linear regression was performed where the severity of airflow limitation was taken as the dependent variable and the factors age, smoking pack-years, SGRQ-C Total %, number of exacerbations in last year, PPD, PI, and CAL were taken as independent variables. Next, the number of exacerbations in the last year was taken as the dependent variable and the factors age, smoking pack-years, SGRQ-C Total %, PPD, PI, and CAL were taken as independent variables. In a separate analysis, another multiple linear regression was conducted for the number of exacerbations in the last year, with the severity of airflow limitation with age, smoking pack-years, SGRQ-C Total %, and periodontal severity as independent variables. The logistic regression analysis was conducted with the presence of hospitalizations during the last year as the dependent variable and PPD, PI, and CAL. Pearson’s correlation was performed to identify the correlation between numerical variables. A two-tailed *p*-value of <0.05 was considered statistically significant.

## 3. Results

**Figure 1:**
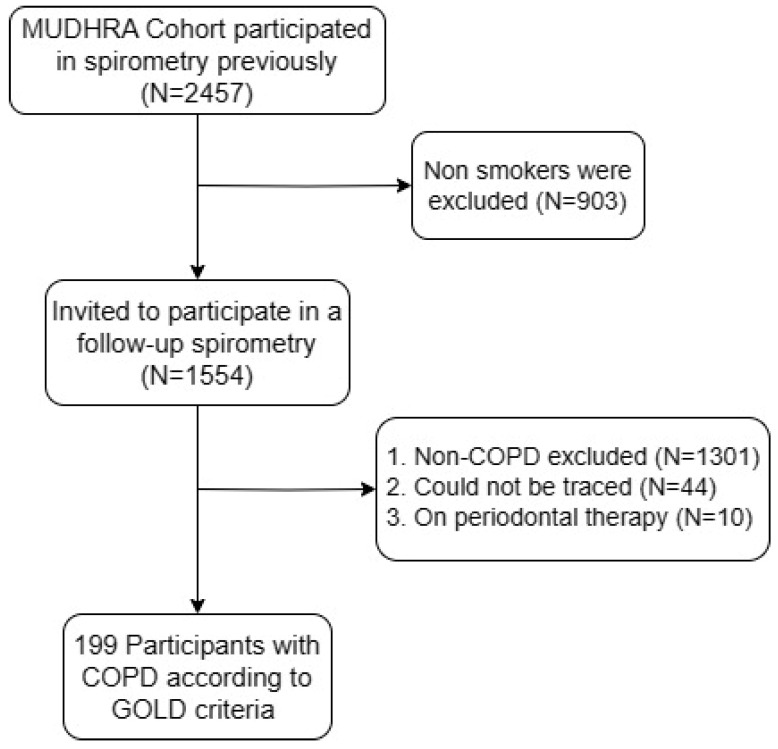
Flowchart of the study participants from the MUDHRA cohort.

This study encompassed a cohort of 199 male subjects, all smokers with confirmed COPD as determined by spirometry (Figure 1). The participants had an average age of 64.47 ± 8.8 years, with a majority involved in farming activities. Among them, 151 were beedi smokers, 32 were cigarette smokers, and 16 smoked both types of tobacco, with average pack-years of smoking at 45 ± 71.5. A detailed breakdown of demographic and study-related variables is provided in Table 1.

### 3.1. Respiratory and Clinical Parameters

Post-bronchodilator spirometry variables were as follows: FVC % predicted at 73.8 ± 20, FEV_1_ % predicted at 53.3 ± 19, and FEV_1_/FVC ratio at 56.3 ± 10. According to the GOLD grading system, 22 subjects were categorized as grade 1, 75 as grade 2, 88 as grade 3, and 14 as grade 4 for airflow limitation. The mean SGRQ-C Total score was 34.6 ± 15, while the predicted mean six-minute walk distance percent was 79.6 ± 13. Acute exacerbation of COPD within the preceding year was recorded in 193 subjects, with 67 of them requiring hospitalization due to exacerbation (Table 1).

### 3.2. Periodontal Severity and COPD Exacerbations

**Figure 2:**
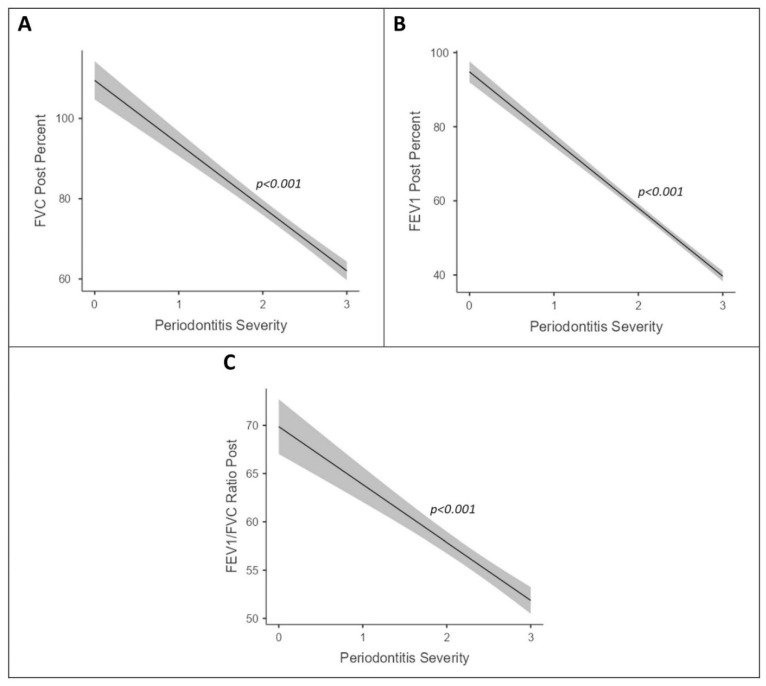
Association between lung function parameters (post-bronchodilator FVC percent predicted (**A**), FEV_1_ percent predicted (**B**), and FEV_1_/FVC ratio (**C**)) and periodontitis severity after adjusting for smoking (pack-years).

**Figure 3:**
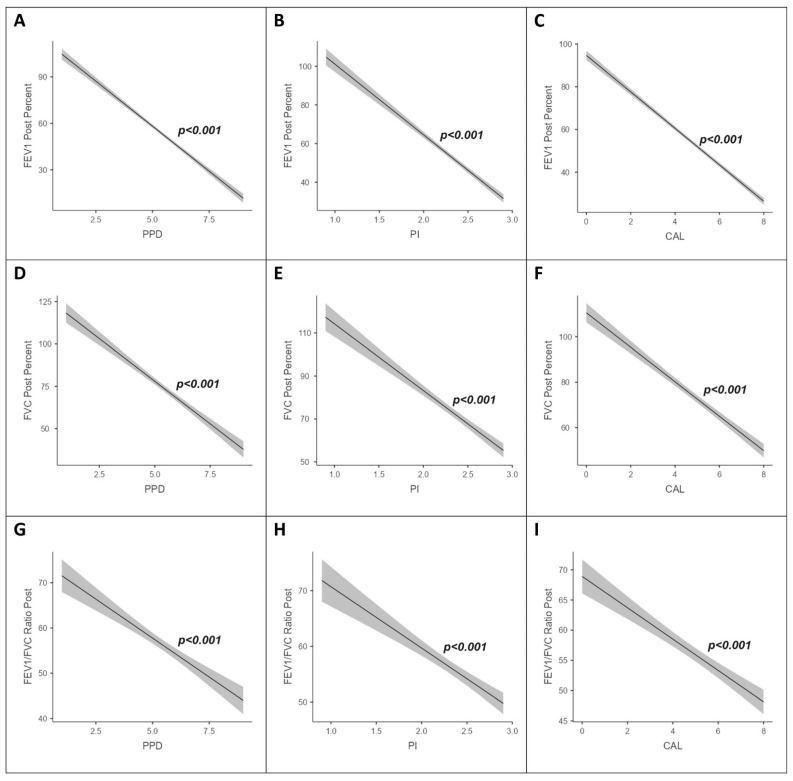
Association between lung function parameters (post-bronchodilator FEV_1_ percent predicted, FVC percent predicted, and FEV_1_/FVC ratio) and periodontal parameters (PPD, PI, and CAL) after adjusting for smoking (pack-years). (**A**) (post-bronchodilator FEV_1_ percent predicted and PPD), (**B**) (post-bronchodilator FEV_1_ percent predicted and PI), (**C**) (post-bronchodilator FEV_1_ percent predicted and CAL), (**D**) (post-bronchodilator FVC percent predicted and PPD), (**E**) (post-bronchodilator FVC percent predicted and PI), (**F**) (post-bronchodilator FVC percent predicted and CAL), (**G**) (post-bronchodilator FEV_1_/FVC ratio and PPD), (**H**) (post-bronchodilator FEV_1_/FVC ratio and PI), and (**I**) (post-bronchodilator FEV_1_/FVC ratio and CAL)

Figure 2 shows the association between lung function parameters (post-bronchodilator FEV_1_ percent predicted, FVC percent predicted, and FEV_1_/FVC ratio) and periodontitis severity, which showed that an increase in periodontitis severity and lung functions followed a decreasing trend even after adjusting for smoking (in pack-years), which was statistically significant (*p* < 0.001). Figure 3 shows the association between lung function parameters (post-bronchodilator FEV_1_ percent predicted, FVC percent predicted, and FEV_1_/FVC ratio) and periodontal parameters (PPD, PI, and CAL), which showed that an increase in periodontal parameters and lung functions followed a decreasing trend even after adjusting for smoking (in pack-years), which was statistically significant (*p* < 0.001).

Table 2 (Model 1) presents regression analysis results, showing the impact of predictors on the number of exacerbations experienced in the last year. For each additional pack-year of smoking, the predicted number of exacerbations increases by approximately 0.0028 (*p* = 0.023). Higher GOLD stages are significantly associated with more exacerbations. For each unit increase in the GOLD stage, the predicted number of exacerbations increases by approximately 1.15 (*p* < 0.001). Compared to the reference level of “no” severity, individuals with “moderate” severity have a predicted decrease of approximately 0.47 exacerbations (*p* = 0.029).

Table 2 (Model 2) presents the regression analysis results assessing the impact of various predictors on the GOLD stage, a measure of airflow limitation severity. Compared to the reference level of “no” severity, individuals with “severe,” “mild,” and “moderate” severity have significantly higher predicted GOLD stages (*p* < 0.001). For each exacerbation experienced in the last year, the predicted GOLD stage increases by approximately 0.30889 (*p* < 0.001).

### 3.3. Periodontal Indices and COPD Severity

**Figure 4:**
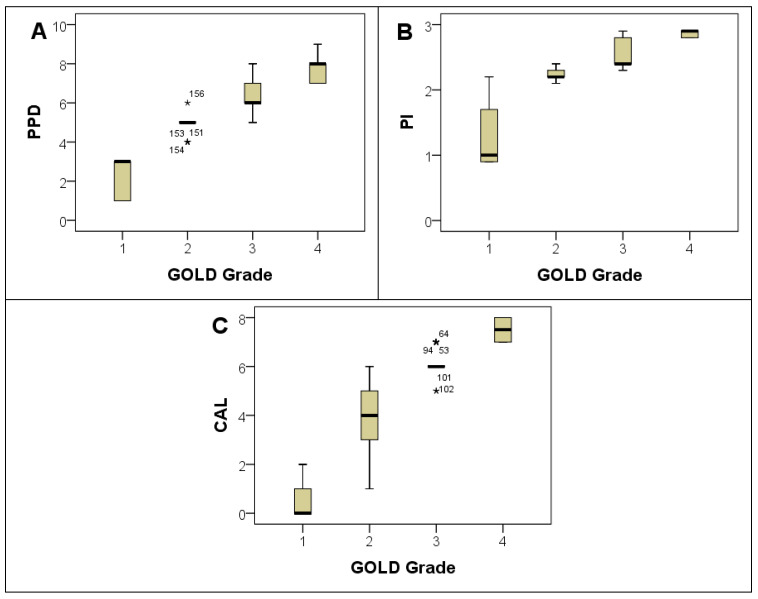
Box plots for PPD (**A**), PI (**B**), and CAL (**C**) with GOLD Grade groups. PPD: Probing Pocket Depth; PI: Plaque Index; CAL: Clinical Attachment Loss. Note: Extreme outliers are marked with a star (★) on the boxplot.

**Figure 5:**
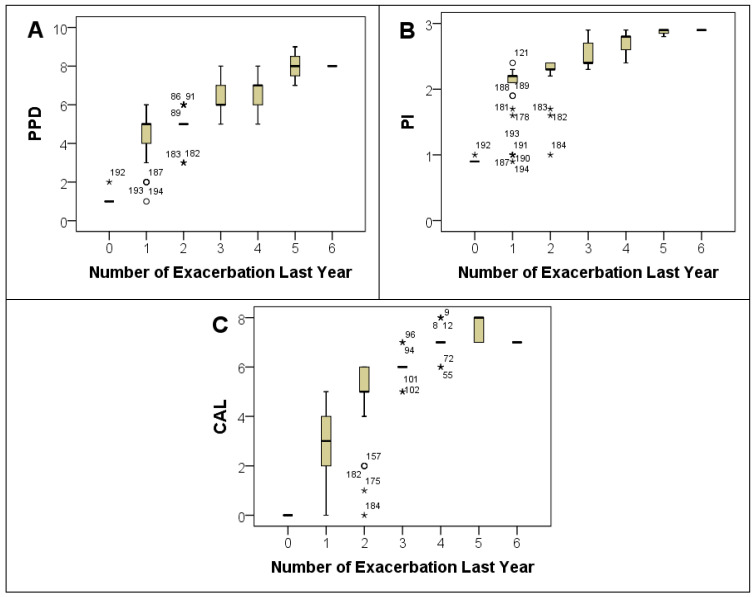
Box plots for PPD (**A**), PI (**B**), and CAL (**C**) with the number of exacerbations during the last year. PPD: Probing Pocket Depth; PI: Plaque Index; CAL: Clinical Attachment Loss. Note: Extreme outliers are marked with a star (★) on the boxplot and mild outliers with a circle (O).

Mean values for periodontal indices were 5.4 ± 1.5 for probing pocket depth (PPD), 2.3 ± 0.5 for plaque index (PI), and 4.8 ± 2.1 for clinical attachment loss (CAL). Comparing these indices across different COPD severity grades revealed a dose–response relationship, where higher GOLD grades were associated with greater mean scores of PPD, PI, and CAL (Table 3 and Figure 4A–C). There was a notable increase in the average tooth loss with increasing COPD severity. Participants with GOLD grade 4 COPD had the highest average tooth loss (7.8 ± 1.1; *p* < 0.001) and higher periodontal severity (*p* < 0.001) (Table 1). Furthermore, a clear trend emerged when assessing the relationship between the mean levels of these indices and the number of exacerbations within the past year. Subjects with a higher number of exacerbations demonstrated higher mean values for PPD, PI, and CAL, as illustrated in Figure 5.

### 3.4. Correlations and Regression Analyses

**Figure 6:**
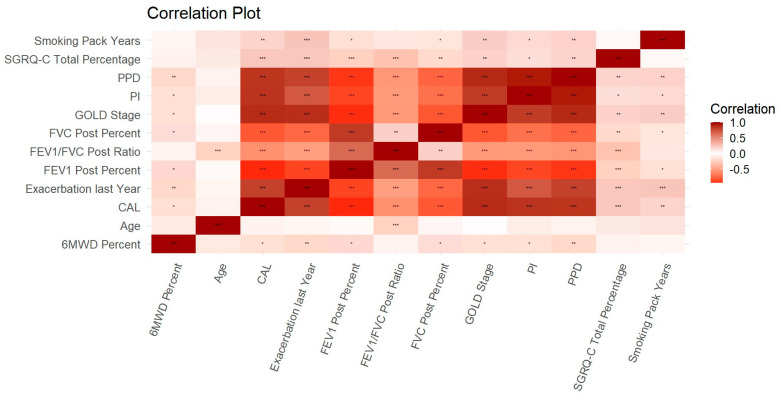
Plot showing the level of correlation between the variables. PPD: Probing Pocket Depth; PI: Plaque Index; CAL: Clinical Attachment Loss. Note: * = *p* < 0.05, ** = *p* < 0.01, *** = *p* < 0.001

Significant correlations emerged among the studied variables. PPD, PI, and CAL exhibited significant correlations with the six-minute walk distance (6MWD), spirometry variables (FVC %Pred, FEV_1_ %Pred, and FEV_1_/FVC ratio), SGRQ-C Total score, and the number of exacerbations in the previous year, as elucidated in Figure 6. Linear regression analyses with PPD, PI, and CAL as independent variables demonstrated an independent association with both the GOLD grading of COPD and the number of exacerbations in the last year (Table 4). Coefficients revealed that for every unit increase in PPD, PI, and CAL, the GOLD grading increased by 0.288, 0.718, and 0.193, respectively. Similarly, for the number of exacerbations during the last year, a unit increase in PPD, PI, and CAL correlated with an increase in the number of exacerbations by 0.115, 0.041, and 0.109, respectively. Additionally, a logistic regression analysis demonstrated the independent association of PPD, PI, and CAL with the presence of hospitalization in the past year (Table 5). The adjusted odds ratios (ORs) for PPD, PI, and CAL were 1.29 (95% CI: 1.03–1.62), 3.04 (95% CI: 1.28–7.2), and 1.26 (95% CI: 1.06–1.49), respectively. Multiple linear regression analysis revealed a significant independent association between COPD (GOLD stages) and periodontitis (PPD, PI, and CAL) (*p* < 0.001), while no significant independent association was found between smoking in pack-years and periodontitis. In univariate linear regression, a significant association was observed between smoking and periodontitis (PPD, PI, and CAL) (Table 6).

## 4. Discussion

This cross-sectional study involved 199 COPD subjects, recruited from a tertiary care university teaching hospital-led community study (MUDHRA cohort) in South India, the patients were categorized into GOLD Grades 1 to 4, and periodontitis was evaluated by assessing the PPD, PI, and CAL, as well periodontal disease severity based on CDC. Three key clinical outcomes in relation to chronic periodontitis were assessed: severity of airflow limitation, acute exacerbations of COPD (AECOPD) in the previous year, and hospitalizations due to AECOPD. We found a strong correlation between increasing periodontitis severity and lower FEV_1_, FVC, and FEV_1_/FVC ratio even after adjusting for smoking (in pack-years). Furthermore, a strong correlation between increasing periodontal parameters (PPD, PI, and CAL) and lower FEV_1_, FVC, and FEV_1_/FVC ratio even after adjusting for smoking (in pack-years) was observed. We observed that smoking pack-years, poorer quality of life, periodontitis (moderate severity), and higher GOLD stage are associated with a higher predicted number of exacerbations. Additionally, periodontal severity, along with the number of exacerbations in the last year, have a significant impact on the predicted GOLD stage (airflow limitation severity). Other factors such as age and smoking pack-years do not show statistically significant effects on the GOLD stage. Exacerbation frequency was significantly associated with GOLD grades (*p* < 0.0012), with Grade 1 patients experiencing fewer exacerbations compared to Grade 4 patients. Hospitalization rates also showed a significant association (*p* = 0.0272), with higher proportions of hospitalizations in higher GOLD-grade patients. Health-related quality of life, as assessed by SGRQ-C, demonstrated significant differences (*p* = 0.0021), indicating worsening quality of life with increasing GOLD grades. However, exercise tolerance, measured by the six-minute walk distance, did not reach statistical significance (*p* = 0.0641) between different GOLD groups. The study revealed statistically significant associations between CP severity, as defined by GOLD grades, and various periodontal parameters. Probing pocket depth (PPD) exhibited substantial differences across GOLD grades (*p* < 0.0011), with Grade 4 patients having the highest mean PPD (6.2 ± 0.7 mm) compared to other grades. Plaque index (PI) demonstrated a significant association with GOLD grades (*p* < 0.0011), indicating an increase in plaque accumulation with worsening CP severity. Clinical attachment loss (CAL) displayed a significant association with GOLD grades (*p* < 0.0011), highlighting increasing attachment loss (≥5 mm) as CP severity advanced with an increase in GOLD grades. Linear regression analysis indicated that PPD, PI, and CAL were significantly associated with airflow limitation along with exacerbation frequency. For every unit increase in PPD, PI, and CAL, the GOLD grading increased by 0.288, 0.718, and 0.193, respectively. For every unit increase in PPD, PI, and CAL, the number of exacerbations experienced in the previous year increased by 0.115, 0.041, and 0.109 respectively. PPD, PI, and CAL had an independent and significant association with hospitalization due to acute exacerbation of COPD. Due to interactions among variables in multiple regression analysis, the effect of a predictor variable can change direction when other predictor variables are included in the model. The negative coefficient for PI in Model 4 (Table 4) indicates that when PI is considered alongside other variables in this model, its impact on GOLD grading and COPD exacerbations appears to be negative.

Chronic obstructive pulmonary disease (COPD) represents a global health challenge with high mortality rates. Recently, the intersection of COPD with other inflammatory conditions, particularly periodontitis, has garnered significant attention. Numerous studies have explored the relationship between COPD and various clinical markers of periodontal disease, including tooth loss [29,30,31,32,33]. A meta-analysis by Shi et al. underscored the association between COPD and key periodontal parameters such as gingival bleeding, poor oral hygiene, periodontal pocket formation, clinical attachment loss, and tooth loss [34]. Additionally, the link between inflammatory mediators such as prostaglandin E2 (PGE2) and airway obstruction severity in COPD, as identified by Chen et al., suggests a potential mechanism for shared inflammation pathways [35,36,37].

Common inflammatory mediators, such as PGE2 and IL-1, in both COPD and periodontitis, highlight potential cross-talk between these conditions. PGE2’s role in triggering inflammation in stable COPD patients suggests its involvement in disease progression [38]. In periodontitis, PGE2 and IL-1 play pivotal roles in the destruction of periodontal tissues. This shared involvement of inflammatory pathways could provide a mechanistic basis for the observed association between the two conditions [39].

The convergence of risk factors in COPD and periodontitis raises intriguing questions. Both conditions share risk factors such as tobacco use, advanced age, reduced immunity, and socio-economic status [40,41,42]. However, it is crucial to distinguish between causation and acceleration, as these risk factors may not directly initiate the diseases but could accelerate their progression [22]. The socio-economic connection is noteworthy, with Shen et al. reporting higher incidences of periodontal diseases and hospitalizations among COPD patients with low socio-economic status [43]. This shared burden underscores the importance of addressing disparities to mitigate disease progression. Notably, our observations revealed a significant increase in tooth loss with growing severity of COPD (GOLD grading). A cohort study by Barrot et al. further supported this, emphasizing the significance of tooth loss or fewer remaining teeth in the oral cavity as a notable risk factor for COPD, with edentulous patients having a 2.28-fold higher risk [44].

Persistent periodontal health issues consistently correlate with reduced lung function and worsened COPD outcomes [45]. While establishing causality requires further exploration, these findings underscore the critical role of maintaining good oral health in COPD management and the preservation of respiratory function. Periodontal indices, including probing pocket depth, bleeding on probing, and loss of attachment, displayed negative correlations with key pulmonary function parameters, including FEV1% predicted and FEV1/FVC ratio. Moreover, these indices exhibited positive correlations with COPD Assessment Test (CAT) scores, indicating an association between periodontal problems, increased COPD severity, and reduced patient quality of life [46]. Another study highlighted significant associations between clinical attachment level (CAL) and FEV1% predicted in COPD subjects. Additionally, the study identified the prevalence of specific oral pathogens, such as Porphyromonas gingivalis (Pg), in individuals with COPD, revealing a significant negative association between Pg content and FEV_1_% predicted. This reaffirms the interconnectedness of periodontal health, oral pathogens, and lung function [47]. Among the Chinese population, a higher likelihood of severe periodontal disease was observed in individuals with more severe COPD. Various periodontal indices, including probing depth (PD), attachment loss (AL), plaque index (PI), alveolar bone loss, and the number of teeth, significantly correlated with all stages of COPD, and PI emerged as a strong predictor for COPD [48]. Another study observed an association between COPD and periodontal health, revealing that individuals with COPD had significantly worse CAL, PD, and oral hygiene index (OHI). A significant negative correlation was found between lung function (FEV_1_ values) and various periodontal indices, confirming that deteriorating periodontal health corresponded to increased lung obstruction in COPD patients [30]. In a study by Zhou, a significant increase in FEV_1_ and FEV_1_/FVC was observed in COPD patients undergoing periodontal therapy during a follow-up of 1 and 2 years, compared to control groups without periodontal treatment [49]. This emphasizes the pivotal relationship between periodontitis and COPD, as treatment of periodontal diseases shows significant improvement in lung functions of COPD patients. In our study, we observed a significant negative correlation between periodontal disease severity and lower FEV_1_, FVC, and FEV_1_/FVC ratio. Similarly, we observed a negative correlation between periodontal parameters (PPD, PI, and CAL) and lower FEV_1_, FVC, and FEV_1_/FVC ratio.

Our study reveals that severe COPD patients show stronger associations with periodontitis, specifically with respect to plaque index (PI), clinical attachment loss (CAL), and probing pocket depth (PPD). Among these, PI emerges as a critical factor, influencing key COPD outcomes, including the severity of airflow limitation, number of exacerbations in the previous year, and hospitalizations due to AECOPD, emphasizing the pivotal role of plaque as a primary contributor. The aspiration of oral bacteria along with respiratory pathogens, influencing adherence and colonization, suggests a potential route for lung disease progression [50,51]. Integrating periodontal therapy and oral hygiene measures might reduce the respiratory infection burden, indirectly influencing COPD progression [49]. When considering the positive effects of periodontal treatment on COPD, several mechanisms come into play. Firstly, periodontal therapy has been demonstrated to reduce the concentration of various inflammatory biomarkers, potentially leading to a less severe inflammatory response in COPD patients [39,52]. Secondly, the removal of dental plaque may decrease the colonization of the oral mucosa by bacteria frequently isolated in dental and respiratory specimens from COPD patients, particularly during exacerbations [50,53]. However, it is crucial to note that some authors have documented worsened respiratory symptoms due to bacterial contamination from aerosols generated during dental treatment with ultrasonic instruments [54,55]. As preventative measures, avoiding air polishing, employing large-volume evacuation suction, and utilizing antimicrobial pre-procedural mouthwash are recommended [55].

Smoking plays a pivotal role as a shared modifier, influencing both periodontitis and COPD. The intricate relationship among smoking, periodontitis, and COPD highlights the multifaceted nature of disease interactions [56]. Our analysis reveals positive associations between PPD, PI, CAL, pack-years, and COPD acute exacerbations in the previous year. Smoking cessation emerges as a crucial intervention, potentially ameliorating risk factors for both conditions. Notably, although age and smoking pack-years did not exhibit significant associations with the severity of airflow limitation, their impact might manifest over more extended periods or in interaction with other factors [57,58]. The variability in the significance of certain predictors across models emphasizes the importance of considering multiple variables to gain a comprehensive understanding of disease progression [59].

In our study, we observed an independent association of periodontal disease with COPD and not with smoking (pack-years). Si et al. [48] observed that PI was a significant risk factor for COPD in current smokers (odds ratio (OR) 8.28; 95% CI: 2.36 to 29.0), former smokers (OR: 5.89; 95% CI 2.64 to 13.1), and non-smokers (OR 2.46; 95% CI: 1.47 to 4.10) (adjusted for age, sex, occupation, educational level, and smoking status). Hyman et al. [60] observed that current smokers with ≥4 mm mean attachment loss (MAL) had significantly higher odds for COPD (OR: 3.71; 95% CI: 1.74 to 7.89) (adjusted for age, gender, race/ethnicity, history of hypertension, history of heart attack, dental visit within 1 year, BMI, year smoked, and family income). However, among former or non-smokers, no significant association between MAL and COPD was observed. Scannapieco et al. [61] observed that subjects with MAL ≥ 3.0 mm had higher odds of COPD (odds ratio 1.45; 95% CI 1.02 to 2.05) than those having MAL < 3.0 mm (adjusted for gender, age, race, education, income, dental treatment history, alcohol consumption, diabetes status, and smoking status). Harland et al. [62] observed that the association between COPD and smoking was stronger for men with periodontitis (OR 2.45; 95% CI 1.37 to 4.37) than those without periodontitis (OR 1.64; 95%CI 0.91 to 2.94) (adjusted for age, number of present teeth, body mass index, alcohol intake, occupation, hypertension, and diabetes). Takeuchi et al. [41] observed a significantly increased risk of COPD among severe periodontitis subjects (RR = 3.51; 95% CI 1.15 to 10.74) (adjusted for sex, age, occupation, diabetes mellitus, body mass index, physical activity, alcohol intake, smoking intensity, and number of present teeth). Lopen-de-Andres et al. [35] observed that COPD subjects had an increased risk of periodontal disease (OR 1.21; 95% CI 1.12 to 1.30) compared to non-COPD subjects. These diverse findings underscore the complexity of the relationship between COPD, smoking, and periodontitis, with variations observed across different studies and populations.

Adding another layer of complexity, the role of education in disease burden comes into focus. Higher education is associated with a lower likelihood of periodontal diseases. Our study’s alignment with research by Kim et al. underscores the broader socio-demographic context surrounding COPD and periodontitis [63]. These findings accentuate the need for holistic healthcare strategies that address both clinical and socio-economic factors.

In the evolving landscape of research, the link with COVID-19 has become a focal point [64,65,66]. Drawing parallels with the established connection between COPD and periodontal disease, we consider the potential oral–lung pathway through which oral infections from periodontitis might heighten susceptibility to COVID-19 infection and/or symptoms [67,68,69]. This connection adds a new dimension to our understanding, emphasizing the intricate interplay between respiratory health and periodontal conditions.

While this manuscript offers valuable insights, our study had several limitations. The cross-sectional design restricts our ability to infer causality from observed associations. Additionally, unaccounted for confounding variables such as alcohol, drug abuse, oral tobacco or betel nut chewing, oral hygiene, brushing duration, hypertension, coronary artery disease, chronic liver disease, autoimmune disease, and cancer were not included in the analysis. Longitudinal studies are warranted to establish temporal relationships and better understand the trajectories of disease progression. Furthermore, the absence of age- and gender-matched controls without COPD limits the comprehensive nature of the analysis. Future research should incorporate these elements to enhance the predictive accuracy of disease progression models.

## 5. Conclusions

In conclusion, our study highlights the intricate connection between periodontitis and COPD (airflow limitations and exacerbation), revealing the impact of periodontitis on the natural course of COPD progression. The identification of these relationships highlights the need for integrated management strategies that encompass oral hygiene measures, smoking cessation, and targeted interventions. The link between inflammatory mediators, risk factors, and clinical implications underscores the importance of interdisciplinary collaboration and personalized healthcare approaches. Implementing proper periodontal therapy, addressing smoking cessation, and adopting oral hygiene measures can potentially delay disease progression by reducing COPD exacerbations. However, further longitudinal trials are necessary to elucidate the causal relationship between COPD and periodontitis, thus uncovering the precise underlying mechanisms governing this intriguing interplay. As we move forward, acknowledging the dynamic interplay between periodontitis and COPD is essential for optimizing patient care and advancing our understanding of complex disease interactions. Physicians and dentists should monitor and increase awareness among COPD patients to maintain oral hygiene for the prevention of periodontal diseases and their effect on the progression of COPD.

## Figures and Tables

**Table 1:** Demographic characteristics and distribution of variables of the study subjects.

	Total (N = 199)	GOLD Grade				
		1 (N = 22)	2 (N = 75)	3 (N = 88)	4 (N = 14)	*p-*Value
Age	64.5 ± 8.8	59.2 ± 11.2	66.8 ± 8.5	64.3 ± 8.1	61.4 ± 4.6	0.001 ^1^
Occupation						<0.001 ^2^
Farmer	72 (36.2)	11 (50.0)	30 (40.0)	22 (25.0)	9 (64.3)	
Retired farmer	104 (52.3)	9 (40.9)	37 (49.3)	57 (64.8)	1 (7.1)	
Others	23 (11.6)	2 (9.1)	8 (10.7)	9 (10.2)	4 (28.6)	
Smoking Type						0.037 ^2^
Beedi	151 (75.9)	21 (95.5)	53 (70.7)	67 (76.1)	10 (71.4)	
Cigarette	32 (16.1)	0 (0.0)	19 (25.3)	11 (12.5)	2 (14.3)	
Both	16 (8.0)	1 (4.5)	3 (4.0)	10 (11.4)	2 (14.3)	
Smoking Pack-Years	39.9 ± 35	27.5 ± 19	33.3 ± 24	47.4 ± 45	48.1 ± 25	0.016 ^1^
FVC % Pred ^#^	73.8 ± 20	105.2 ± 7	83.6 ± 11	61.8 ± 12	46.9 ± 18	<0.001 ^1^
FEV_1_ % Pred ^#^	53.3 ± 19	88.5 ± 6	64.1 ± 8	39.8 ± 6	25.4 ± 3	<0.001 ^1^
FEV_1_/FVC Ratio ^#^	56.3 ± 10	66.3 ± 3	60.5 ± 7	50.7 ± 9	53.3 ± 8	<0.001 ^1^
SGRQ-C Total %	34.6 ± 15	27.6 ± 16	32.3 ± 15	38.8 ± 13	32.1 ± 13	0.002 ^1^
6MWD %	79.6 ± 13	82.8 ± 11	81.0 ± 13	78.8 ± 13	72.1 ± 16	0.064 ^1^
Tooth loss	5.6 ± 1.1	4.0 ± 0.7	5.0 ± 0.1	6.2 ± 0.6	7.8 ± 1.1	<0.001 ^1^
Tooth present	23.0 ± 2.1	27.1 ± 1.5	23.6 ± 1.1	21.9 ± 0.9	20.2 ± 1.1	<0.001 ^1^
Periodontal Severity						<0.001 ^2^
None	14 (7.0)	14 (63.6)	0 (0)	0 (0)	0 (0)	
Mild	28 (14.1)	8 (36.4)	20 (26.7)	0 (0)	0 (0)	
Moderate	50 (25.1)	0 (0)	48 (64.0)	2 (2.3)	0 (0)	
Severe	107 (53.8)	0 (0)	7 (9.3)	86 (97.7)	14 (100)	
Presence of Exacerbation in Last Year						<0.001 ^2^
No	6 (3.0)	6 (27.3)	0 (0.0)	0 (0.0)	0 (0.0)	
Yes	193 (97.0)	16 (72.7)	75 (100.0)	88 (100.0)	14 (100.0)	
Number of Exacerbations						
Mean ± SD	2.3 ± 1	0.9 ± 0.6	1.3 ± 0.5	3.1 ± 0.6	4.8 ± 0.7	<0.001 ^1^
Median (IQR)	2 (1–3)	1 (0–1)	1 (1–2)	3 (3–3)	5 (4–5)	<0.001 ^1^
Presence of Hospitalization in Last Year						0.027 ^2^
No	132 (66.3)	16 (72.7)	56 (74.7)	55 (62.5)	5 (35.7)	
Yes	67 (33.7)	6 (27.3)	19 (25.3)	33 (37.5)	9 (64.3)	

^1^ Kruskal–Wallis test, ^2^ Pearson; ^#^ Spirometry (Post Bronchodilator values); FVC: Forced Vital Capacity; FEV_1_: Forced Expiratory Volume in 1 s; % Pred: Percent Predicted; 6MWD: 6-min walk distance; SGRQ-C: St. George’s Respiratory Questionnaire Scores.

**Table 2:** Multiple linear regression analysis for risk for severity of airflow limitation (GOLD grading) and number of exacerbations.

	Model 1	Model 2
Age	0.002 (−0.003–0.007)	−0.007 (−0.017–0.003)
Smoking Pack-Years	−0.000 (−0.001–0.001)	0.003 (0.0002–0.005) *
SGRQ_C Total Percentage	−0.002 (−0.005–0.001)	0.006 (−0.000–0.012) *
Periodontal Severity:		
Severe	1.273 (1.043–1.502) ***	0.130 (−0.436–0.696)
Mild	0.556 (0.355–0.757) ***	−0.344 (−0.761–0.072)
Moderate	0.808 (0.618–0.998) ***	−0.474 (−0.899-−0.048) *
Exacerbation	0.309 (0.250–0.368) ***	-
GOLD Stage	-	1.159 (0.938–1.381) ***

* = *p* < 0.05, *** = *p* < 0.001; Dependent Variable: Model 1—GOLD stage, Model 2—Number of exacerbations during the last year; SGRQ-C: St. George’s Respiratory Questionnaire Scores; GOLD: Global Initiative for Obstructive Lung Disease.

**Table 3:** Comparison of means and medians of the variables PPD, PI, and CAL between GOLD gradings of airflow limitation.

	Total (N = 199)	GOLD Grade				*p*-Value
		1 (N = 22)	2 (N = 75)	3 (N = 88)	4 (N = 14)	
PPD						
Mean ± SD	5.4 ± 1.5	2.3 ± 0.9	4.9 ± 0.3	6.2 ± 0.7	7.7 ± 0.7	<0.001 ^1^
Median (IQR)	5 (5–6)	3 (1–3)	5 (5–5)	6 (6–7)	8 (7–8)	<0.001 ^2^
PI						
Mean ± SD	2.3 ± 0.5	1.3 ± 0.5	2.2 ± 0.1	2.5 ± 0.2	2.9 ± 0.1	<0.001 ^1^
Median (IQR)	2.3 (2.2–2.5)	1 (0.9–1.7)	2.2 (2.2–2.3)	2.4 (2.4–2.8)	2.9 (2.8–2.9)	<0.001 ^2^
CAL						
Mean ± SD	4.8 ± 2.1	0.6 ± 0.8	4.0 ± 1.3	6.2 ± 0.5	7.5 ± 0.5	<0.001 ^1^
Median (IQR)	6 (4–6)	0(0–1)	4 (3–5)	6 (6–6)	7.5 (7–8)	<0.001 ^2^

^1^ Kruskal–Wallis test, ^2^ Pearson; PPD: Probing Pocket Depth; PI: Plaque Index; CAL: Clinical Attachment Loss; GOLD: Global Initiative for Obstructive Lung Disease.

**Table 4:** Multiple linear regression analysis: risk factors for severity of airflow limitation (GOLD grading of airflow limitation) and number of exacerbations during last year.

Risk Factors for Severity of Airflow Limitation	Model 1	Model 2	Model 3	Model 4
Age	0.0002 (−0.005–0.005)	0.0003 (−0.0050–0.0056)	0.0006 (−0.0045–0.0057)	−0.001 (−0.005–0.004)
Smoking Pack-Years	0.00003 (−0.001–0.001)	0.0001 (−0.0013–0.0014)	0.0001 (−0.0012–0.0014)	0.000 (−0.001–0.001)
SGRQ-C Total Percentage	−0.0004 (−0.003–0.003)	0.0001 (−0.0031–0.0032)	−0.002 (−0.0051–0.0011)	−0.002 (−0.004–0.001)
Number of Exacerbations in last year	0.263 (0.207–0.319) ***	0.351 (0.298–0.403) ***	0.281 (0.221–0.340) ***	0.177 (0.119–0.236) ***
PPD	0.288 (0.241–0.334) ***	-	-	0.280 (0.196–0.364) ***
PI	-	0.718 (0.574–0.861) ***	-	−0.319 (−0.584–−0.054) **
CAL	-	-	0.193 (0.157–0.228) ***	0.132 (0.091–0.174) ***
**Risk Factors for Number of Exacerbations during Last Year**	**Model 1**	**Model 2**	**Model 3**	**Model 4**
Age	−0.012 (−0.022–−0.001) *	−0.011 (−0.021–−0.001) *	−0.012 (−0.022–−0.002) *	−0.011 (−0.021–−0.001) *
Smoking Pack-Years	0.003 (0.000–0.005) *	0.003 (0.000–0.005) *	0.003 (0.000–0.005) *	0.003 (0.000–0.005) *
SGRQ-C Total Percentage	0.007 (0.001–0.013) *	0.007 (0.001–0.013) *	0.006 (−0.000–0.012)	0.004 (−0.002–0.010)
GOLD Stage	1.173 (0.924–1.422)	1.349 (1.146–1.551) ***	1.114 (0.879–1.348) ***	0.892 (0.598–1.186) ***
PPD	0.115 (−0.015–0.244) ***	-	-	0.316 (0.112–0.519) **
PI	-	0.041 (−0.304–0.385)	-	−0.911 (−1.500–−0.323) **
CAL	-	-	0.109 (0.021–0.196) *	0.161 (0.062–0.260) **

* = *p* < 0.05, ** = *p* < 0.01, *** = *p* < 0.001; Dependent variable: Number of exacerbations during last year (0 to 6 exacerbations); Model 1: PPD, Model 2: PI, Model 3: CAL, and Model 4: Combined PPD, PI, and CAL; CI: Confidence Interval; SGRQ-C: St. George’s Respiratory Questionnaire Scores; PPD: Probing Pocket Depth; PI: Plaque Index; CAL: Clinical Attachment Loss; GOLD: Global Initiative for Obstructive Lung Disease.

**Table 5:** Logistic regression analysis: risk factors for hospitalization during last year.

Model	Model 1	Model 2	Model 3
Variable	OR (CI)	OR (CI)	OR (CI)
Age	0.98 (0.94–1.01)	0.97 (0.94–1.01)	0.98 (0.95–1.02)
Smoking Pack-Years	1.0 (0.99–1.02)	1.0 (0.99–1.02)	1.0 (0.99–1.02)
SGRQ-C Total %	1.0 (0.98–1.02)	1.0 (0.97–1.02)	0.99 (0.97–1.02)
PPD	1.29 (1.03–1.62) *	-	-
PI	-	3.04 (1.28–7.2) *	-
CAL	-	-	1.26 (1.06–1.49) **

* = *p* < 0.05, ** = *p* < 0.01; Dependent Variable: the presence of hospitalization during last year; OR (CI): Odds Ratio (95% Confidence Interval). Model 1: PPD, Model 2: PI, and Model 3: CAL; SGRQ-C: St. George’s Respiratory Questionnaire Scores; PPD: Probing Pocket Depth; PI: Plaque Index; CAL: Clinical Attachment Loss.

**Table 6:** Multiple linear regression analysis to assess the independent association of smoking (pack-years) and COPD (GOLD stages) with periodontitis (PPD, PI, and CAL).

Variable	Model 1	Model 2	Model 3
Age	−0.002 (−0.013–0.008)	−0.001 (−0.005–0.002)	0 (−0.016–0.016)
Smoking Pack-Years	0 (−0.002–0.003)	0 (−0.001–0.001)	−0.001 (−0.004–0.003)
GOLD Stage			
2–1	2.678 (2.375–2.981) ***	0.969 (0.866–1.072) ***	3.339 (2.875–3.802) ***
3–1	3.984 (3.688–4.279) ***	1.251 (1.15–1.352) ***	5.589 (5.137–6.042) ***
4–1	5.44 (5.024–5.857) ***	1.578 (1.436–1.72) ***	6.874 (6.237–7.511) ***

*** = *p* < 0.001; Dependent Variable: Model 1: PPD, Model 2: PI, and Model 3: CAL. PPD: Probing Pocket Depth; PI: Plaque Index; CAL: Clinical Attachment Loss; GOLD: Global Initiative for Obstructive Lung Disease.

## Data Availability

All data generated or analyzed during this study are included in this published article and are available from the corresponding author upon reasonable request. The data is not publicly available due to privacy and ethical restrictions.
